# Current status of artificial intelligence methods for skin cancer survival analysis: a scoping review

**DOI:** 10.3389/fmed.2024.1243659

**Published:** 2024-04-22

**Authors:** Celine M. Schreidah, Emily R. Gordon, Oluwaseyi Adeuyan, Caroline Chen, Brigit A. Lapolla, Joshua A. Kent, George Bingham Reynolds, Lauren M. Fahmy, Chunhua Weng, Nicholas P. Tatonetti, Herbert S. Chase, Itsik Pe’er, Larisa J. Geskin

**Affiliations:** ^1^Vagelos College of Physicians and Surgeons, Columbia University, New York, NY, United States; ^2^Department of Dermatology, Columbia University Irving Medical Center, New York, NY, United States; ^3^Jacobs School of Medicine and Biomedical Sciences, University at Buffalo, Buffalo, NY, United States; ^4^The Data Science Institute, Columbia University, New York, NY, United States; ^5^Department of Biomedical Informatics, Columbia University, New York, NY, United States; ^6^Department of Computational Biomedicine, Cedars-Sinai Medical Center, Los Angeles, CA, United States; ^7^Cedars-Sinai Cancer, Cedars-Sinai Medical Center, Los Angeles, CA, United States; ^8^Department of Systems Biology, Columbia University, New York, NY, United States; ^9^Department of Computer Science, Columbia University, New York, NY, United States

**Keywords:** artificial intelligence, skin cancer, oncology, machine learning, deep learning, supervised learning, unsupervised learning, natural language processing

## Abstract

Skin cancer mortality rates continue to rise, and survival analysis is increasingly needed to understand who is at risk and what interventions improve outcomes. However, current statistical methods are limited by inability to synthesize multiple data types, such as patient genetics, clinical history, demographics, and pathology and reveal significant multimodal relationships through predictive algorithms. Advances in computing power and data science enabled the rise of artificial intelligence (AI), which synthesizes vast amounts of data and applies algorithms that enable personalized diagnostic approaches. Here, we analyze AI methods used in skin cancer survival analysis, focusing on supervised learning, unsupervised learning, deep learning, and natural language processing. We illustrate strengths and weaknesses of these approaches with examples. Our PubMed search yielded 14 publications meeting inclusion criteria for this scoping review. Most publications focused on melanoma, particularly histopathologic interpretation with deep learning. Such concentration on a single type of skin cancer amid increasing focus on deep learning highlight growing areas for innovation; however, it also demonstrates opportunity for additional analysis that addresses other types of cutaneous malignancies and expands the scope of prognostication to combine both genetic, histopathologic, and clinical data. Moreover, researchers may leverage multiple AI methods for enhanced benefit in analyses. Expanding AI to this arena may enable improved survival analysis, targeted treatments, and outcomes.

## Introduction

Skin cancer is the most common cancer among patients in the United States (1). Over 9,500 people are diagnosed daily, and two people die hourly (2–4). Melanoma, the deadliest skin cancer, (3) accounts for most patient mortality. Epidemiological and clinical investigations improved documentation of skin cancer incidence and prevalence, increasing discussion on prevention and detection. Literature has recognized the paramount importance of early detection and management for skin cancer and the potential for assistance by artificial intelligence (AI) tools at this stage (5). However, monitoring with survival analysis, along with discovery of survival markers are greatly needed for clinical prognostication.

Survival analysis assesses the outcome of time prior to an event of interest (e.g., death, treatment response, disease recurrence, or relapse) and may identify survival markers. Survival analysis in skin cancer research has leveraged univariate and multivariate analyses of national survey databases (6–8). While these analyses elucidated clinical and demographic associations, they are limited by patient reporting and cannot feasibly include multimodal (genetic, histopathologic, and clinical) data. Despite the benefits of focused multimodal cohort survival analyses, significant methodological barriers exist to revealing new insights beyond those via standard statistical methods. For example, innovative multimodal survival analysis time frames pose significant logistical barriers. Leveraging branches of AI may facilitate such research on survival. AI systems possess potential to assist in all stages of research to clinical care: from genomic alteration identification to even clinician-focused workflow tools (9).

Supervised and unsupervised machine learning (ML), deep learning, and natural language processing are AI methods transforming survival analysis. ML automates and scales statistical processes to discover relationships that humans alone cannot find. Four major areas within ML are discussed in this review. Supervised ML makes use of labeled data (i.e., cases where an outcome of interest is known) to find patterns that predict outcomes. Unsupervised ML is used for unlabeled data (i.e., no known outcome of interest) to find structure within data (e.g., to find similar groups or clusters) and previously unknown associations (10). Deep learning is comparatively newer and finds its own data representations, removing much of the need for feature engineering (11). Lastly, natural language processing (NLP) may operate as a form of deep learning built on text data by using neural networks (e.g., human ways of thinking) to find representations of text that form basic, quantifiable understandings of it.

Introduction of AI methods in oncologic research may transform the field, possibly enhancing mechanistic underpinnings of disease, therapeutic target discovery, synergistic treatment regimens, and guidance for clinical decision-making (12–14). With skin cancer rates rising, innovative research leveraging AI is essential (1). Use of AI within cutaneous oncology research has flourished, with studies investigating classification, detection, medical record extraction, risk identification, prediction, and prognosis (12). Applications include tools diagnosing skin cancer using clinical photographs and patient phone applications to track and manage their cancer care (15, 16). AI may augment existing understanding of cutaneous oncology pathogenesis, clinical classification, and prognostication.

In this scoping review, we present publications exemplifying possibilities for AI to innovate skin cancer research, particularly in survival analysis. An advanced search of PubMed was conducted to survey the primary literature from inception to June 11, 2023, using terms related to survival analysis, skin cancer, and AI (Supplemental material), yielding 16 publications. Publications were screened with inclusion and exclusion criteria by multiple investigators (CS, EG, GR), resolving conflicts by discussion and following the guidelines set by the Preferred Reporting Items for Systematic reviews and Meta-Analyses extension for Scoping Reviews (PRISMA-ScR). Criteria for inclusion involved articles incorporating AI in survival analysis for skin cancer patients; original investigations (not reviews); English articles; accessible articles online; no repeated articles. Criteria for exclusion encompassed articles not being original AI investigations on the topic of skin cancer survival analysis; review articles; articles not in English; non-accessible articles online; repeated articles. Two publications were excluded for not utilizing AI methods. Publications were then subject to critical review, with results contextualized within the AI method matrix for skin cancer survival analysis (Table 1). We discuss various types of AI, examples of their application within the field of oncology, and highlight methodology from each publication.

**Table 1:** Summary of included literature on artificial intelligence applied to skin cancer survival analysis.

Article short citation (Author, Year, Journal)	Form of AI	Type of skin cancer(s) Studied	Type of AI data inputs	Source of data	Primary survival outcome(s) investigated	Name of performance metric	Final reported performance metric (numerical)	Limitations discussed
Trincado et al. 2016 (17), Genome Medicine	Supervised learning	Melanoma	RNA sequencing and clinical data; breast tumors according to estrogen receptor (ER) status and melanoma tumors with proliferative and invasive phenotypes	The Cancer Genome Atlas	Tumor staging and clinical outcome	AUC	Logistic model trees (LMT) for each tumor type and stage class – the mean accuracy of the models in terms of AUC is 0.783	Lack of validation on independent cohorts
Wang et al. 2019 (18), Medical Science Monitor	Supervised learning	Melanoma	Long noncoding RNA (lncRNA), microRNA (miRNA) and mRNA	The Cancer Genome Atlas, Gene Ontology database, Kyoto Encyclopedia of Genes and Genomes pathway	Survival	Cox Regression	–	Not discussed
Su et al. 2021 (19), Molecular Cancer Therapeutics	Supervised learning	Melanoma	mRNA expression	The Cancer Genome Atlas	Overall and disease-free survival	Median mRNA expression	Higher expressions of PLK1 and NOTCH1 correlated with worse survival (*p* < 0.001)	Lack of validation in *in vivo* models
Failmezger et al. 2020 (20), Cancer Research	Supervised learning	Melanoma	Topological tumor graphs (TTG)	The Cancer Genome Atlas	Degree of lymphocytic infiltration and overall survival	Cox Regression	–	Lack of access to independent clinical cohorts
Wilson et al. 2021 (21), Artificial Intelligence Medicine	Supervised learning	Melanoma	Gene expression and miRNA expression	University of California at Santa Cruz (UCSC) Xena	Survival status (Dead or Alive)	C-index	MKCox = 0.640	Not discussed
Yang et al. 2018 (22), International Journal of Oncology	Unsupervised learning	Melanoma	Long-coding RNAs (lncRNAs)	The Cancer Genome Atlas	Kaplan-Meiei survival analysis	AUROC	AUROC = 0.816	Limited sample size; sample heterogeneity
Jonckheere and Van Seuningen 2018 (23), Journal of Translational Medicine	Unsupervised learning	Skin cancer (and other cancers)	*MUC4* expression	The Cancer Genome Atlas, Cancer Cell Line Encyclopedia	Overall survival and hazard ratio	AUROC	AUROC MUC4/16/20 = 0.8272	Inadequate statistical power
Yang et al. 2021 (24), PLOS One	Unsupervised learning	Melanoma	Primary tumor (T), regional lymph nodes (N), distant metastasis (M), age (A), and sex (S)	Surveillance, Epidemiology, and End Results Program (SEER) of the National Cancer Institute	Survival time (in months), SEER cause-specific death classification variable, compared to AJCC staging	C-index	C-index = 0.7865	Bias secondary to death certificate errors; the need for a large dataset to obtain robust estimates of survival
Comes et al. 2022 (25), Scientific Reports	Deep learning	Melanoma	Whole-slide histological images (WSIs)	Clinical Proteomic Tumor Analysis Consortium Cutaneous Melanoma (CPTAC-CM) public database then validated on Istituto Tumori “Giovanni Paolo II” in Bari, Italy	1-year disease free survival	AUC	Best predictive classification performances were obtained in terms of median AUC and accuracy with values of 0.695 and 0.727%, respectively	Relatively small size of the analyzed datasets
Johannet et al. 2021 (26), Clinical Cancer Research	Deep learning	Melanoma	Whole slide image (WSI) analysis of metastatic melanoma tissue	Interdisciplinary Melanoma Cooperative Group (IMCG) database at NYU Langone Health; Vanderbilt University Ingram Cancer Center	Progression free survival	AUC	AUC 0.800 on images from the Aperio AT2 and AUC 0.805 on images from the Leica SCN400	Small sample size
Moore et al. 2021 (27), Scientific Reports	Deep learning	Melanoma	H&E whole slide images	The Cancer Genome Atlas	Disease-specific survival	Automated Digital Tumor-infiltrating lymphocyte Analysis (ADTA) score	ADTA contributed to disease-specific survival prediction (*p* = 0.006)	Reliance of model on pathologists; lack of sentinel lymph node biopsies performed in the cohorts
Chou et al. 2021 (28), Modern Pathology	Deep learning	Melanoma	Whole slide images, % TIF	NYU melanoma database	Recurrence-free survival (RFS) and overall survival (OS)	C-index	% TIL was associated with significantly longer RFS (adjusted HR = 0.92 [0.84–1.00] per 10% increase in % TIL) and OS (adjusted HR = 0.90 [0.83–0.99] per 10% increase in % TIL)	Use of a singular data set
Chiu et al. 2021 (29), Annual International Conference of the IEEE Engineering in Medicine and Biology Society (EMBC)	Deep learning	NMSC (SCC and BCC)	Incidence rate of SCC and BCC	Database from the United Network for Organ Sharing (UNOS)	Risk factors highly associated with skin cancer events	AUC, compared CoxTime, DeepSurv, and Cox proportional hazards models	DeepSurv, CoxTime, and Cox proportional hazards model AUCs are 0.772 ± 0.0084, 0.775 ± 0.0105, and 0.756 ± 0.0092	Not discussed
Liestøl et al. 1994 (30), Statistics in Medicine	Deep learning	Melanoma	Surgically-resected samples	University Hospital of Odense, Denmark during 1962–1977	Survival time following radical surgical resection of tumor	Cox Proportional Hazards Model	–	Inclusion of too many parameters

This table summarizes the publications included from our PubMed Advanced search query following critical review with inclusion/exclusion criteria. Publications are summarized, featuring specific details on skin cancer investigated and performance.

## Supervised machine learning

Supervised ML is a subfield of AI that utilizes existing data for future dataset predictions. Following training with known independent variables (i.e., gene expression level) “labeled” with outcomes of interest (e.g., survival time), supervised models can identify patterns and predict outcomes. Ramsdale et al. applied supervised modeling to assess fall risk in older adults with advanced cancer starting chemotherapy (31). After assessing 73 initial features, including number of prior falls and cognitive impairment, the model effectively classified patients as “non-faller” or “faller.” Our search yielded skin cancer publications using similar approaches with RNA-level and tumor architecture data.

A 2016 study by Trincado et al. applied supervised ML to predict clinical outcomes across 12 solid tumor types (17). The study assessed relative abundance of transcripts and applied a multivariate feature selection method on isoforms to generate logistic models for each tumor type and stage, with mean classification performance area under the curve (AUC) of 0.783. The authors applied their model to predict patient survival, analyzing significance with Cox proportional hazards model– a regression assessing effect of several quantitative and categorical risk factors on survival time. Wang et al. similarly used Cox proportional hazards in 2019 to investigate pathogenesis of metastatic melanoma (18). Using bioinformatics data from TCGA (The Cancer Genome Atlas) and other databases, the authors identified differential expression of seven mRNAs, five microRNAs (miRNAs), and six long noncoding RNAs (lncRNAs) correlated with survival in metastatic melanoma patients.

In 2021, Su et al. similarly stratified melanoma patients, though according to expression levels of the serine/threonine kinase PLK1 and transmembrane protein NOTCH1 (19). Cox regression analysis found high expression of both PLK1 and NOTCH1 associated with worse overall survival. They suggested that dual targeting may provide novel means for melanoma treatment. They identified downregulation of multiple melanoma-related pathways and found their top five downregulated genes associated with cancer metastasis.

Beyond RNA expression and protein analysis, supervised ML has also shown promise in mapping the tumor microenvironment architecture. In 2020, Failmezger et al. investigated properties of the tumor microenvironment that may affect melanoma cancer cell targeting (20). Using a novel graph-based algorithm to understand the stromal network, the authors utilized a quantitative morphologic classifier with supervised ML to identify melanoma cancer cells, lymphocytes, and stromal cells. After representing spatial relationships of the three cell types, Cox regression analysis found high stromal clustering and barriers to cancer-infiltrating lymphocytes significantly associated with poor survival.

Supervised ML expands upon previous statistical methods. Wilson et al. (21) exemplified this through application of an alternative Cox loss function for melanoma survival prediction (21). The authors preprocessed gene and miRNA expression data, then training models on training datasets and assessing performance with test sets. Their novel supervised ML approach outperformed other models, highlighting efficiency and flexibility of different supervised algorithms for survival prediction– particularly when integration of various high-throughput data sources is needed.

Overall, these studies show promise for supervised ML in uncovering the genetic basis of melanoma pathogenesis, risk-classifying patients, and predicting tumor behavior based on structure and composition. Current limitations are scarcity of sufficiently populated RNA-sequencing databases and need for validating *in vivo* models. Moreover, a supervised approach requires selection of data and targets with known associations, such that classifying big data can pose a challenge in supervised learning (particularly when compared to unsupervised learning). However, progress in the field shows its value for improved medical decision-making and precision medicine.

## Unsupervised machine learning

Unsupervised ML uses algorithms to cluster and analyze unlabeled datasets, relying on the machine to find previously unknown associations. An unsupervised approach may generate multiple clusters and risk-stratify accordingly. Eckardt and colleagues recently developed a large-scale model with unsupervised ML to isolate four patient clusters using clinical and genetic acute myeloid leukemia data; statistical analysis demonstrated significant differences across various clusters (32). Review of the literature reveals various applications of unsupervised ML to cutaneous oncology survival analysis. These studies identified genetic clusters or introduced patient survival-stratifying attributes.

Several studies applied unsupervised ML to genomic signatures. Yang et al. (22) leveraged a TCGA dataset to identify lncRNAs from samples with melanoma stages I-IV, then using hierarchical clustering and support vector machine analyses to classify the lncRNAs (22). Survival methods included standard Kaplan–Meier analysis (33) yielding a predictive signature of six lncRNAs tested with a validation set. This signature encompassed 720 target genes, corresponding to numerous pathways that may affect melanoma prognosis. The method’s accuracy in risk-stratification of melanoma samples was >80%. This prognostic marker for melanoma risk-classification set the groundwork for further studies to assess the signature’s predictive potential.

Jonckheere and Van Seuningen (23) used unsupervised ML to correlate gene expression with derived prognostic information of *MUC4*, a membrane-bound mucin implicated in multiple cancers (23). This study leveraged online tools to extract *MUC4* Z-score expressions and use hazard ratios and other statistics to generate a list of 187 genes correlated with *MUC4* expression. Two were associated with worse survival in combination with *MUC4*. The large-scale genomic approach enabled authors to overcome prior study limitations of inadequate statistical power, offering potential new biomarkers for targeted treatment. These studies’ prognostic signatures offer promising potential for future applications.

Yang et al. (24) utilized an unsupervised ML approach beyond genetic signatures, leveraging the Ensemble Algorithm for Clustering Cancer Data (EACCD) to integrate additional factors into the traditional TNM (tumor, nodes, metastases) staging system for improved melanoma prognostication (24). Prior studies attempted to augment TNM with Cox regression and tree modeling, but these methods had not clearly risk-stratified patients or led to low prediction accuracy. The authors investigated the clinical meaningfulness of their new clusters via supervised learning, finding that using them as input variables increased prognostic prediction accuracy.

**Figure 1:**
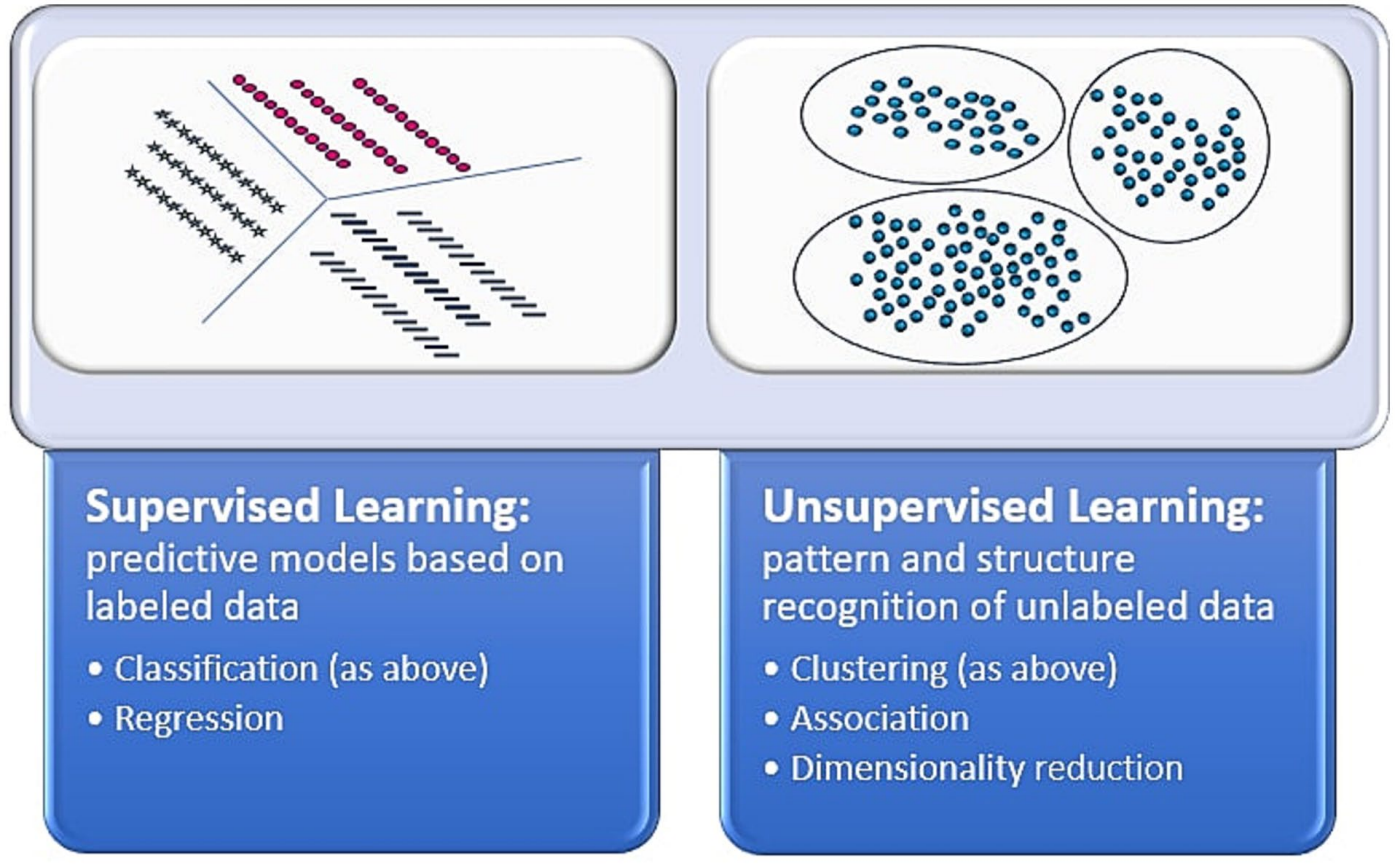
Supervised vs. unsupervised learning. This figure depicts the differences between the artificial intelligence methods of supervised and unsupervised learning (39, 40).

These studies show unsupervised ML’s many applications to skin cancer and survival analysis. Importantly, there are limitations to these analyses, especially related to clinical interpretability of found groups. For instance, there is a possibility that structure may not be found when leveraging unsupervised learning. Still, unsupervised learning has revealed important structures and key associations that aid in understanding of survival and prognostic outcomes. Figure 1 delineates differences between supervised and unsupervised learning.

## Deep learning

Deep learning is a class of multi-layered ML algorithms inspired by the human brain’s structure and function to improve accuracy. Early models were explored by Liestøl et al. (30), who applied neural network– a set of algorithms based on interconnected nodes, or artificial neurons in a layered structure– to commonly used regression models to strengthen survival prediction in melanoma patients (30). The authors found these models moderately improved predictions on survival time for melanoma patients following radical surgical resection, providing the groundwork for modern deep learning and survival methods.

Subsequently, deep learning has been used to better prognosticate and identify genomic alterations in melanoma. Comes et al. (25) aimed to predict 1-year disease-free survival (DFS) in melanoma patients using deep learning applied to hematoxylin and eosin-stained whole slide images (WSIs). The study was limited by a cohort of 43 patients from a public database, though annotations provided by expert pathologists. Still, the authors’ proposed deep learning model extracted quantitative imaging biomarkers from WSIs and demonstrated prognostic power in predicting 1-year DFS, contributing to the investigation of deep learning for prognostication in melanoma patients.

A recent study by Johannet et al. also explored deep learning’s role, correlating melanoma tissue histology with immune checkpoint inhibitor (ICI) response (26). This study investigated whether neural networks could combine important features of melanoma tissue with clinical and demographic data to predict immunotherapy response. A multivariable classifier demonstrated success in separating high and low-risk patients and predicting treatment response, displaying promise for future integration of deep learning tissue digital pathology analysis and clinical/demographic data.

Moore et al. (27) used a different approach of automated digital analysis (ADTA) to study tumor-infiltrating lymphocytes, or TILs, to augment current staging of primary early-stage (i.e., stage II-III) melanomas (27). The authors utilized previously developed deep learning algorithms to tile WSIs and estimate likelihood of TILs in each tile. ADTA score was calculated as the median of “positive” tiles: the likelihood of at least 77.5% TILs in that tile, over the total number of tiles, of all the patient’s images. The authors found that ADTA score correlated with disease-specific survival (DSS) in melanoma and that this approach strengthened predictive value of standard pathology characteristics such as depth and ulceration. Although susceptible to user and cohort variability, this strongly suggests ADTA may exceed performance of standard qualitative TIL assessment for melanoma risk evaluation.

Similarly, a recent study by Chou et al. explored percent of TILs as a predictive measure for melanoma prognosis while offering deep learning as a method to standardize clinician approaches (28). In this retrospective analysis, a neural networks classifier used WSIs to calculate the percentage of TILs in melanoma tissue, which was compared to the manually derived Clark’s grading. This study confirmed the previously established percent TIL threshold of 16.6% and the use of TILs as a prognostic marker, as higher percentages of TILs were associated with both longer recurrence-free survival (RFS) and overall survival. These results demonstrate the value of deep learning in improving TIL counting for melanoma prognosis.

Deep learning methods have been instrumental in predicting risk for other skin cancers. Chiu and colleagues in 2021 utilized two deep neural network-based models (DeepSurv and CoxTime) to predict basal and squamous cell carcinoma risk in heart transplant recipients, comparing their performance to Cox proportional hazards models (29). The authors assessed prediction performance post-heart transplantation, finding DeepSurv and CoxTime models significantly exceeded performance comparatively at every time point. They demonstrated superiority of neural networks in providing improved risk predictions in this patient population.

Overall, these deep learning studies illustrate how ML may solve complex problems, from interpreting images with TILs to integrating various data types into a single model. Deep learning models can be criticized for limitations such as requirement for vast amounts of data and lack of applicability to new data. However, they provide promise in rapidly analyzing various data types and greatly improving current survival methods.

## Natural language processing

Natural language processing (NLP) encompasses computer-based algorithms that transform natural language, such as blocks of text, into usable information for research (34). For example, it may integrate contextual nuances, or word clues, to define necessary words to extract for analysis. GPT-4 is a large language model (LLM) that performs tasks from solving advanced mathematical problems to writing personal essays. NLP may utilize a “rules” approach: user instructing the computer on information to extract; another approach is with machine learning: inputting training data, letting the computer practice/learn, and identifying or extracting learned words or phrases of interest. Amidst an increase in availability of accessible biological and medical population databases, NLP holds promise in potentially eliminating the need for manual review among clinicians and researchers (34).

NLP has been utilized in dermatology and oncology, from synthesis of biopsy reports to extraction of symptoms from patient histories to survival analysis. Yuan in 2021 used NLP to find key cancer characteristics from a cohort >40,000 patients with lung cancer (35). They used NLP to compile structured data (i.e., diagnoses) and unstructured data (narrative notes) to develop a prognostic model to estimate lung cancer survival (AUC = 0.82).

Our cutaneous oncology survival analysis search did not yield NLP publications. However, upon manual search for “natural language processing” and “survival analysis,” we encountered one melanoma NLP study. Yang in 2021 investigated if TILs were an independent prognostic factor for overall survival in primary cutaneous melanoma (36). NLP combed through notes and identified clinical and histopathologic data, performing regression analyses demonstrating brisk TILs significantly associated with improved survival.

The identified lack of NLP survival analysis publications might be due to limitations in searching or NLP-oriented tasks. Many studies use NLP as a data extraction tool for word frequency or isolation. Thus, it is less predominantly featured in survival analysis, though has potential to uncover prognostic indicators.

## Discussion

We reviewed AI’s application to survival analysis for cutaneous malignancies. While AI has expanded its reach within oncology, applications to survival analysis and cutaneous oncology remain limited. Secondly, types of skin cancer and data analyzed were similar. Lastly, several publications leveraged multiple AI branches, with increasing focus on deep learning and less on NLP.

Only 16 publications resulted from our query, with several excluded for lack of relevance. Survival analysis remains a ripe area for multimodal AI application, enabling extraction of pathology and clinico-demographic data to generate predictive models. Few publications may have resulted due to our search’s limitations; we expect an increase as data extraction advances.

Nearly every publication studied melanoma, despite greater prevalence of non-melanoma skin cancers, likely due to melanoma’s mortality burden. Non-melanoma skin cancers are areas for future analysis to elucidate prognostic indicators.

Many publications investigated integrating genetics, clinical data, and/or histology. They used data from similar sources (e.g., TCGA) and similarly reported AUC or C-index. These similarities speak to reliance on large databases and statistical standardization. Additionally, several publications analyzed images with a deep learning approach deconstructing to components, identifying patterns, and mapping results onto current disease understanding. Predictive modeling with images has made significant progress, owing to ease of machine training on thousands of images versus far fewer attributes.

The division between the discussed AI branches is not rigid; researchers might utilize “deep learning” methods but also integrate supervised learning. A multifaceted approach enables researchers to develop more complex algorithms and synthesize disparate data. For example, researchers may use NLP to extract unstructured data from clinical notes, deep learning for histology, and supervised learning for regression toward survival analysis and prognostication. Each approach has benefits and limitations, but together they may enhance modeling potential.

Several investigations illustrate limitations of AI in healthcare (37). LLMs are error-prone, sometimes inconsistently pulling information from records and relying on false generalizations. Other limitations include smaller datasets (potentially over-weighing certain features) or even incomplete and biased datasets, highlighting a need for quality of data inputs to develop algorithms. Disparities in inclusion of skin of color images in datasets poses negative implications for model generation and subsequent performance, biasing models and yielding inequitable results and representation (38). Thus, issues like loss-to-follow-up, note errors, or hospital transfers provide incomplete clinical scenarios; many models had non-excellent performances (AUCs <90%). Relying on new models that incompletely capture clinical situations may have devastating consequences: patients receiving inappropriate treatments or inaccurate prognostication. Thus, critical analysis and data synthesis is essential to AI.

Future investigations may engage with a diversity of cutaneous malignancies, using multiple AI methodologies to leverage benefits and compensate for any weaknesses. Finally, studies may expand beyond survival to integrate quality-of-life analyses.

Overall, this review is an important contribution to increasing literature on AI applications to survival analysis for patients with skin cancer. Innovative applications may reveal unique insights in clinical settings to enable physicians to better assess patient survival and develop targeted treatment.

## Author contributions

CS, EG, and LJG: conception and design of study. CS, EG, OA, CC, BL, JK, GR, and LG: acquisition of data and drafting the manuscript. CS, EG, OA, CC, BL, JK, GR, LF, CW, NT, HC, IP, and LJG: revising the manuscript for important intellectual content. All authors contributed to the article and approved the content for publication.
