# Supplementary material for: Current status of artificial intelligence methods for skin cancer survival analysis: a scoping review
Source: Front Med (Lausanne). 2024 Apr 22;11:1243659. doi: 10.3389/fmed.2024.1243659 (PMC11070520; doi:10.3389/fmed.2024.1243659)
Supplement: Supplementary file 1 [file Data_Sheet_1.docx]

**Supplemental Text:**

The following is the PubMed advanced search query:

("Survival Analysis"[Mesh] OR Analysis, Survival*[tiab] OR Analyses, Survival*[tiab] OR Survival Analyses*[tiab])) AND ("Skin Neoplasms"[Mesh] OR Neoplasms, Skin*[tiab] OR Neoplasm, Skin*[tiab] OR Skin Neoplasm*[tiab] OR Cancer of Skin*[tiab] OR Skin Cancers*[tiab] OR Cancer of the Skin*[tiab] OR Skin Cancer*[tiab] OR Cancer, Skin*[tiab] OR Cancers, Skin*[tiab])) AND ("Artificial Intelligence"[Mesh] OR Intelligence, Artifical*[tiab] OR Computational Intelligence*[tiab] OR Intelligence, Computational*[tiab] OR Machine Intelligence*[tiab] OR Computer Reasoning*[tiab] OR Reasoning, Computer*[tiab] OR AI (Artificial Intelligence)*[tiab])
